# Tracing the decision-making process of physicians with a Decision Process Matrix

**DOI:** 10.1186/s12911-016-0369-1

**Published:** 2016-10-18

**Authors:** Daniel Hausmann, Cristina Zulian, Edouard Battegay, Lukas Zimmerli

**Affiliations:** 1 Department of Psychology, Applied Social and Health Psychology, University of Zurich, Binzmuehlestrasse 14, Box 14, Zurich, 8050 Switzerland; 2 Division of Internal Medicine, University Hospital Zurich, Zurich, Switzerland

**Keywords:** Medical decision-making, Decision process matrix, Information search, Confidence

## Abstract

**Background:**

Decision-making processes in a medical setting are complex, dynamic and under time pressure, often with serious consequences for a patient’s condition.

**Objective:**

The principal aim of the present study was to trace and map the individual diagnostic process of real medical cases using a *Decision Process Matrix [DPM])*.

**Methods:**

The naturalistic decision-making process of 11 residents and a total of 55 medical cases were recorded in an emergency department, and a DPM was drawn up according to a semi-structured technique following four steps: 1) observing and recording relevant information throughout the entire diagnostic process, 2) assessing options in terms of suspected diagnoses, 3) drawing up an initial version of the DPM, and 4) verifying the DPM, while adding the confidence ratings.

**Results:**

The DPM comprised an average of 3.2 suspected diagnoses and 7.9 information units (cues). The following three-phase pattern could be observed: *option generation*, *option verification*, and *final diagnosis determination*. Residents strove for the highest possible level of confidence before making the final diagnoses (in two-thirds of the medical cases with a rating of *practically certain*) or excluding suspected diagnoses (with *practically impossible* in half of the cases).

**Discussion:**

The following challenges have to be addressed in the future: real-time capturing of emerging suspected diagnoses in the memory of the physician, definition of meaningful information units, and a more contemporary measurement of confidence.

**Conclusions:**

DPM is a useful tool for tracing real and individual diagnostic processes. The methodological approach with DPM allows further investigations into the underlying cognitive diagnostic processes on a theoretical level and improvement of individual clinical reasoning skills in practice.

“Patients come to a physician with complaints, and the physician attempts to identify the illnesses responsible for the complaints. […] Treatments can be looked up in reference materials; making the correct diagnosis is more complex.” George Bergus, p. 379 [1].

## Background

Making a diagnosis is essential to clinical medicine and is often a prerequisite for a specific medical treatment. First of all, making a diagnosis is a process, usually termed as a *diagnostic* or *medical decision-making process* [1–4]. Within this decision-making process the physician is collecting relevant information (like signs, symptoms etc.) during history-taking. Additionally, clinical information search usually includes a physical exam, imaging methods and data file retrieval. Whereas an information search is a central aspect in the field of decision-making, options come to the fore regarding underlying cognitive *diagnostic reasoning* or *problem-solving* processes within a clinical framework [3, 5–7]. Regarding the process of how physicians come up with and decide between medical options, several theoretical models have been formulated and tested, like pattern recognition, predicting rules or hypothesis testing [1, 3]. Hypothesis testing, also called the hypothetico-deductive method, is viewed as the predominant approach. Within this approach, first diagnoses are hypothetically generated in a rapid succession based on limited information about the chief complaint. In a second phase these competing suspected diagnoses are then tested or verified with further information collected during the decision-making process [1]. In general the subdivision of a decision-making process into several phases seems common, ranging from a simple two-phase to a multi-phase structure [8, 9]. With regard to the goal of a medical examination, the evaluation of suspected diagnosis and the search for more information are seen as a separate step, ideally resulting in a final diagnosis, followed by the selection of an appropriate medical treatment [1, 9].

But making a diagnosis means also dealing with diagnostic uncertainty [10–14]. Symptoms can be more or less distinct, or different illnesses may have similar symptoms. In our understanding, the value of a searched information (e.g. the degree of temperature, pain etc.) can be interpreted by a physician as more or less feasible or even as a subjective probability speaking for or against a specific option (suspected diagnosis) at this time. The evidence accumulation approach and threshold models support this assumption of subjective confidences [15, 16] of single information units or integrated information. Making a final diagnosis and administering medical treatment is likely to take place if the probability of one suspected diagnosis is above a specific threshold [16–18].

For an initial conclusion, generating and testing hypotheses (options), searching for information (cues), and dealing with uncertainty (levels of confidence) are constituent parts of a medical decision-making process [1].

### Decision-making processes within naturalistic medical settings

In practice, medical decision-making processes are often *complex*, *dynamic* and under *time pressure*, especially in emergency departments [12, 19–24]. Physicians’ diagnostic decision-making in an emergency setting poses a challenge for several reasons: patients’ complaints represent a wide range of potentially acute life-threatening conditions which need to be defined and treated or excluded as fast as possible; their physical conditions can abruptly deteriorate; and physicians usually have limited knowledge of the patient’s personal history [24]. Furthermore, time as well as the existing resources are limited, since other patients are waiting in the next examining room [24]. Therefore, physicians have to define and verify suspected diagnoses and possible differential diagnoses efficiently in order to initiate adequate treatment in a timely manner. Under these naturalistic conditions, the diagnostic process may appear heterogeneous and, on an *individual* basis, result in different amounts of incorporated information and numbers of mentioned options, as well as in different subjective certainty about the suspected and final diagnoses and the accuracy of the final diagnosis.

An outstanding methodological challenge is still the non-disruptive, preferably real-time capturing of the course of the most important environmental and introspective variables of an individual engaged in specific tasks during his or her entire decision-making process. As Lipshitz et al. [21] concluded, multiple methodological approaches should be included to counteract the limitations of a single process-tracing method [25]. The critical question is how these different aspects such as generating and testing relevant options, processing relevant information and dealing with uncertainty can be measured with a combination of situational and mental protocols and be integrated into one picture that maps the diagnostic process of an individual physician [5].

Up to now we have had no standardized method for tracing individual decision-making processes of real medical cases. But this very issue, the tracing and visualizing of individual decision-making processes would be invaluable for several reasons: for verifying models about medical decision-making and problem-solving models (theory), providing individual feedback to physicians (education), and optimizing structural processes within medical institutions (e.g., cost-efficiency [6]).

### Combined methodological approach for assessing the diagnostic process

In this article we want to introduce a method of tracing diagnostic decisions with a Decision Process Matrix. In the decision-making literature a classical decision matrix is a frequently used starting point to display decisional situations under uncertainty, to better understand a decision problem, and to raise the rationality of a decision or solution [26–29]. Usually options or alternatives are placed in rows, criteria in columns, and numerical values or rated elements in cells, all displayed together in a table. In a reduced form, two options (A or B) are described with two criteria, for example, the difficulties and consequences for each option. In economics, matrices often use opposed dimensions with this four-fold-pattern, e.g., price and service, or availability and quality, etc. A decision matrix can become more complex when it includes more options and/or more than two relevant criteria. It is a widespread way to display and solve different kinds of decision-making problems in diverse fields and applications. For example, decision matrices are commonly used in multi-criteria decision analysis problems, e.g., when making design decisions in engineering, etc. It can be adapted to similar forms, for example, a belief decision matrix with belief distributions for each element [30], etc. For all forms, the requirement is that it completely assess the decisional situation with all its options, criteria and values [29]. Furthermore, all criteria have to be well-defined, and a method (e.g., rating system) has to be found to measure concrete values with regard to each criterion and option. Finally, a determination needs to be made about whether the criteria should be weighted or not and which decision rule should be applied. Therefore, a classical decision matrix is a simple method to quickly provide a good overview for the decisional situation and potential solutions.

**Fig. 1:**
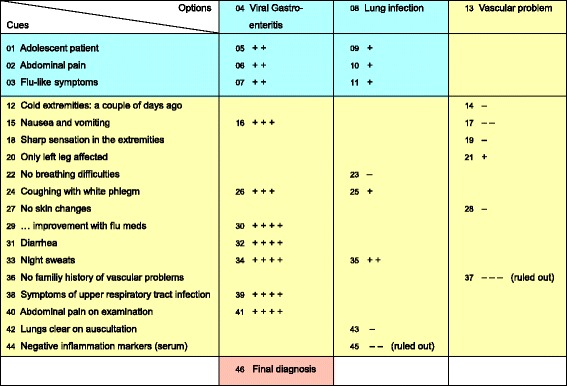
One example (real medical case) of a Decision Process Matrix (DPM) showing a typical three-phase pattern in an 18 year old male patient with a four-day history of fever and abdominal pain. Numbers 01–46 represent the order in which options, cues and confidences occurred. *1. Option generation phase* (from 01–11, highlighted in *blue*): three information (cues) 01 “adolescent patient”, 02 “abdominal pain”, and 03 “flu-like symptoms” consecutively led to two suspected diagnoses (options), namely 04 “Viral Gastroenteritis” and 08 “Lung infection”, each with the corresponding confidence of “quite probable” (05–07) for “Viral Gastroenteritis”, and “probable” (09–11) for “Lung infection”. This first phase follows the sequence cue(s) – mentioned option(s) – corresponding confidence(s) at the beginning of the diagnostic process. *2. Option verification phase* (from 12–45, highlighted in *yellow*): With 15 further cues (including two physical examinations (40 and 42) and laboratory analysis (44)), the suspected diagnoses were verified in detail, resulting in an increase in the confidence in “Viral Gastroenteritis” and an exclusion of “Lung infection” in step 45. Due to information 12 “Cold extremities a couple of days ago”, an additional suspected diagnosis was mentioned (13 “Vascular problem”) with a confidence rating of “*thinkable*” (14) at the outset, which also ended up being excluded at step 37. This second phase follows the sequence (single) cue – confidence (according to that cue and one of the specific mentioned options before). Please note that there does not have to be a confidence value for each mentioned cue and option. *3. Determination of the final diagnosis* (46, highlighted in *red*). After step 44, the laboratory analysis, the resident ruled out “Lung infection” and finally decided in step 46 on a final diagnosis of “Viral Gastroenteritis” and initiated the appropriate treatment. Following the membership functions of 13 verbal probability expressions [39], the following labels have been used for confidence ratings: “-4” = *practically impossible*, “-3” = *improbable*, “-2” = *doubtful*, “-1” = *thinkable*, “0” = *possible*, “+1” = *probable*, “+2” = *quite probable*, “+3” = *very probable*, and “+4” = *practically certain*

For a more dynamic and naturalistic investigation of the diagnostic process we suggest extending this conventional matrix in two directions: first, adding the order of occurrence of the cues, options and values with consecutive numbering and, second, replacing values by rating the subjective confidence in an option at a specific point in time. We then call this expanded map a *Decision Process Matrix [DPM]* (see also Fig. 1). The output of this standardized procedure results in an extended matrix with process information about suspected diagnoses or hypotheses (options), relevant information (cues), and course of confidence ratings over time. Confidence ratings (instead of values) were introduced to address the uncertainty within the diagnostic decision process [14]. By using the consistent measurement of the course of subjective confidence, factors such as the significance and coping with uncertainty from the beginning to the end of the diagnostic process can be explored and linked to different model assumptions (e.g. evidence accumulation [15] approaches and confidence threshold concepts [16, 17]).

### How to validate the mapping of individual processes in a naturalistic medical setting?

When introducing a new method like DPM, how can we be sure the individual diagnostic processes are assessed in an objective and valid manner? On the one hand, each type of variable must be recorded as independently and as objectively as possible. On the other hand, the individual perspective of the diagnosing physician has to be considered too. To incorporate both aspects (objective recording and subjective processing) we chose a semi-structured procedure for assessing the DPM. Observer and physician both have to agree in the final DPM.

For a DPM one should formulate a minimum of at least two claims: A recorded medical case, visualized within a DPM has to be comprehensible (at least to other medical experts) and should bear comparison with a re-examination of the underlying medical case (e.g., inspection of the medical record or further treatment). Such comparisons will have greater statistical and explanatory power if recorded medical cases are as heterogeneous as possible. With respect to the miscellaneous variables within the DPM, it is advantageous as well to have a wide range of correct options (a broad range of medical diagnoses, including mono-morbid and multi-morbid diagnoses), requiring different amounts of information (easy and complex medical cases) and having varying confidence ratings (cases with low and high certainty), as well as a certain pressure to determinate a final diagnosis. For all these aspects, the emergency setting seemed to be the ideal testing field, all but as a robustness test for assessing and validating the DPM in clinical practice.

### Research questions

Within this article we propose a method for tracing essential parts of individual diagnostic decisions with a DPM. To test the feasibility of this method, we applied the method for real, heterogeneous medical cases in an emergency department. *Question 1*: Is it possible to assess and map even heterogeneous, complex and dynamic decisions with a DPM? *Question 2*: How accurately can DPM be assessed? To test the internal validity of this method, subjective diagnostic outcomes (final diagnoses) have been checked for plausibility and accuracy by an expert (re-diagnosis). *Question 3*: Is it possible to map real medical cases in a way that allows comparison with existing theoretical considerations? To test the external validity of this method, the results of the DPM were compared to common decision-making models, like hypothesis testing and evidence accumulation. Specifically, does the DPM map processes as described in the literature about sub-phases (e.g., option generation and option verification) and confidence ratings (e.g., such as increasing confidence for final diagnoses)? *Question 4*: Last but not least, do individual and contextual factors such as experience, time pressure or perceived stress have an effect on the number of options, amount of information, and the level of the rated confidences? For example, expert physicians are often observed collecting less information than novice physicians when making a diagnosis, but are much more likely to make the correct diagnosis [1]. Expert physicians might be expert in knowing which data to collect as well as which data are irrelevant to the diagnostic task.

## Methods

### Setting

The University Hospital Zurich is a teaching hospital that provides primary, secondary, and tertiary care for a region with a population of approximately 390,000 people. 36,000 patients are treated annually at the emergency department [ED]. The present study was conducted with residents in internal medicine. During each working shift the medical staff for all non-surgical emergencies consists of three residents in internal medicine, most of whom have more than two years of postgraduate clinical training. Residents are supervised by one attending physician who is present in the emergency unit between 8 a.m. and 12 a.m. and who is on call between 12 a.m. and 8 a.m.

Patients are received and triaged in surgical or nonsurgical emergencies by specially trained nurses and assigned to one of eighteen emergency treatment units (examining rooms), awaiting the initial contact with a resident of the emergency department. Residents are trained to conduct the diagnostic process in accordance with the following course of action: medical history-taking [MHT], physical examination [PE], ordering of imaging and laboratory tests [I&LT], and integrating all information into a final diagnosis and/or specific differential diagnoses [FD/DD], usually with a certain time interval between I&LT and FD/DD.

### Participants

A total of 11 residents agreed to record their decision-making processes. The residents (45 % female, 55 % male) had an average of 41 months (23–69 months) of experience in internal medicine and a mean age of 34 years (*SD* = 3), ranging from 31–39 years.

### Medical cases

A master’s degree candidate in Psychology accompanied the residents during several different working shifts for two months, with a mean of 1.7 medical cases per resident and shift. Patients who were in an imminent life-threatening situation, under the age of 18, had obvious alcohol abuse, drug abuse or mental illness or who did not speak German have been not included. Furthermore, only medical cases in which the resident had no advance information about the patient were included. Data from a total of 55 medical cases was recorded with a mean of 5.0 cases per resident recorded (*SD* = 1.3, ranging from 3–7). The patients (51 % female) had a mean age of 51 years (ranging from 18–83). Common reasons for presentation were dyspnea (18 %), abdominal pain (18 %), headache (13 %), chest pain (11 %) and other symptoms (40 %). The average duration of the patient history-taking was 18 min (ranging from 10–30 min). The study was approved by the local ethics committee (EK–1628/2009). All of the patients as well as the residents gave a written informed consent.

### Procedure for drawing up the Decision Process Matrix [DPM]

The student accompanied the resident in the emergency room and observed the interaction with the patient throughout the entire diagnostic process. For each medical case, a DPM was drawn up according to a semi-structured technique, following four steps (see Table 1):

**Table 1:** Study overview and the four steps of assessing a Decision Process Matrix

Step	Activities	Assessing	Method	Who	Timepoint
1	Observing and recording relevant medical information	Cues	Taking notes	Student	Throughout the entire diagnostic process
2	Assessing options in terms of suspected diagnoses	Options	Open question	Student & resident	Immediately after the initial contact with patient (after MHT/PE)
3	Drawing up an initial version of the DPM	Raw version of DPM	Transferring cues and options	Student	Between MHT/PE and waiting for I&LT
4	Verifying and completing the DPM and finally adding of confidence ratings	Final versions incl. numbering and confidence ratings	Thinking aloud, reference scale	Resident & student	Directly after the discharge of the patient

The naturalistic decision-making process of 11 residents and a total of 55 medical cases were recorded in a emergency department. For each medical case a Decision Process Matrix [DPM] was drawn up according to a semi-structured technique following steps 1–4. For each step the activities, assessed parts of the DPM, method used, person(s) involved, and timepoint during the diagnostic process are indicated. Resident and student both had to agree in the final DPM. Further abbreviations: MHT, medical history-taking; PE, physical examination; I&LT, imaging and laboratory tests

1. *Observing and recording information (cues)*: While following and observing the physician all the time, the student recorded the flow of information throughout the entire diagnostic process. The student took notes of all verbal communication as well as the observed information search behavior of the physician in detail, including MHT, PE, I&LT, and the further search for additional information via the medical literature and/or an electronic database (e.g., detailed information about potential diagnoses).
2. *Assessing options in terms of suspected diagnoses*: Immediately after the initial contact with the patient (usually after the MHT and PE), the student asked the resident for all possible options (initially mentioned diagnosis and all further suspected diagnoses) he or she had during the entire diagnostic process. The student asked the physician with the help of an open question (“Which options have been considered?”).
3. *Drawing up a first version of the DPM*: During the time interval between the MHT and PE and waiting for the results of the I&LT the student merged cues (step 1) and options (step 2) into a raw version of a DPM. The student first condensed related information into short and meaningful information units (see example of cues in Fig. 1). Those meaningful cues were listed vertically and the mentioned options horizontally in the matrix, all in accordance with their chronologically observed and mentioned appearance. Generally, the initial formulation of cues and options in this draft DPM corresponded at this point fairly well to the underlying diagnostic process of the resident.
4. *Verifying the DPM, adding numbering and confidence rating*: As soon as possible (usually directly after the discharge of the patient), student and resident conferred in a separate room and completed the DPM together. The student presented the raw version of the matrix (step 3) and the resident then verified cues and options in the draft. The resident added (if necessary) additional relevant cues and/or suspected diagnoses, which may have emerged after the I&LT or while consulting the literature. Then, after having checked for all relevant cues and options in the DPM, the physician recapitulated the entire diagnostic process and added place marker for the subsequent confidence ratings. Then, the student and resident together fixed the final order of appearance (adding consecutive numbers from 1 to x). To finish, the resident added confidence ratings (instead of place markers) with the help of a 9-point Likert scale ranging from “-4“(*practically impossible*) to “+4” (*practically certain*).

All four steps described above (see Table 1) resulted in a DPM. One example of a real medical case assessed as a DPM is shown in Fig. 1.

It is important to note that cues, options and confidence rating were assessed separately with different methodological approaches: searched information while taking notes, suspected diagnoses with an open question, sequences with a thinking aloud approach while recapitulating the diagnostic process, and confidence ratings with a reference scale. A strict semi-structured approach had been chosen; however, physician (as involved) and student (as observer) both had to agree in the final DPM.

### Post-hoc verification of medical cases according to accuracy

To check for internal validity, a senior physician with more than 15 years of experience in internal and emergency medicine, respectively, checked the final diagnoses of the 55 medical cases for plausibility and accuracy. On the basis of all of the medical records including the I&LT he reanalyzed each medical case and arrived at an independent diagnosis. The senior physician rated the confidence of his final diagnosis with the same 9-point Likert scale ranging from “-4“(*practically impossible*) to “+4” (*practically certain*). The re-diagnoses and confidence ratings of the senior physician were seen as a reference and were then compared to those of the residents (percentage of concordant final diagnoses) and confidence rating (numerical differences in the ratings of the final diagnoses in the DPM).

### Accessing contextual variables

The three contextual variables “subjective sense of time pressure”, “perceived stress”, and “case experience” were recorded as soon as possible after the discharge of the patient with a short questionnaire for the resident, measured with three separate items: “was there a sense of time pressure?”, “was there a sense of stress induced by any person?”, and “have you encountered similar cases within your clinical practice?”, with three possible response categories each (*“yes”*, *“no”*, or *“don’t know”*). In addition, a rating for similarity was assessed if “case experience” was affirmed, measured with a 6-point Likert scale ranging from “1“(*not similar*) to “6” (*very similar*).

### Statistical analysis

First, the DPM were analyzed descriptively according to the arithmetic means, standard deviations, and ranges of the three most important resulting variables: number of searched cues and mentioned options as well as the values of the rated confidence (initial and final rating of an option within the DPM). Second, all DPM were systematically checked for sub-phase patterns. Third, the accuracy of the final diagnoses was calculated both as a percentage of concordant final diagnoses and as the mean difference between confidence ratings of the final diagnoses of senior physician minus resident, anticipating a high consensus between residents and senior physician in the determination of the final diagnoses as well as low numerical differences in the ratings. Fourth, the effect of the physicians’ general experience in internal medicine (in months) on the resulting variables was calculated with correlations according to Pearson [*r*] and their case experience (degree of similarity) with correlations according to Spearman [*rs*]. All analyses were performed using IBM SPSS Statistics 23 for Windows. Furthermore, effect sizes (*d*’) were calculated according to Cohen.

The study was independent of external funding sources.

## Results

### Number of suspected diagnoses and amount of information within the diagnostic process

On average the 55 Decision Process Matrices [DPM] comprised 3.2 suspected diagnoses (options) (*SD* = 1.8; 1–9) and 7.9 information units (cues) (*SD* = 2.9; 3–17). In all but one case, the following sub-phase pattern was observed: a *first phase*, where a sequence of one or more cues (1–4) led to the first or multiple options mentioned (1–6), each with indication of the corresponding confidence ratings (see sequence of numbers in the example of Fig. 1); *a second phase,* where a series of further cues (1–14) were searched in sequential order, resulting in a confidence rating for single cues and options; and *a third phase,* where the resident determined the final diagnosis or differential diagnoses. These three phases correspond to the three stages of *option generation*, *option verification* and *final decision* (see the Discussion section).

On average, the stage of option generation (*phase 1*) consisted of 2.3 suspected diagnoses (*SD* = 1.4; 1–6) emerging on the basis of 1.9 cues (*SD* = 0.8; 1–4). In the stage of option verification (*phase 2*), 6.1 further cues were accumulated (*SD* = 2.8; 1–14). Furthermore, in nearly half of all cases (45 %) additional options were introduced and verified during the second phase (*M* = +0.9; *SD* = 1.4; 1–7).

### Subjective certainty and the relationship between confidence ratings and inclusion or exclusion of diagnoses

The suspected diagnoses in phase 1 were initially rated with a mean of 1.76 (*SD* = 0.81; “-2” = *doubtful* to “+3” = *very probable*). In 87 % of all 55 medical cases, the final diagnosis was one of the suspected diagnoses in phase 1. Those final diagnoses were rated in phase 3 with a mean of 3.56 (*SD* = 0.71; “+1” = *probable* to “+4” = *practically certain*). 67 % of them were rated with the highest possible confidence value (“+4” = *practically certain*). All the suspected diagnoses that were excluded ended with an average rating of −2.50 in phase 2 (*SD* = 2.08; “-4” = *practically impossible* to “+2” = *quite probable*). 54 % of them were given the lowest possible confidence value (−4 = *practically impossible*).

### Accuracy of the final diagnoses and confidence concordance

The final diagnoses of the residents were concordant with the diagnoses of the senior physician in 93 % (51 out of 55 medical cases). For three cases (of the same resident), one has to assume that the DPM remained incomplete since some of the relevant cues and/or options were not recorded until the end of the diagnostic process: for two of them, the (concordant) final diagnoses were written down in the medical record, but not recorded in the DPM, and in one case the final diagnosis was mentioned in the DPM as a suspected diagnosis, but the patient was finally diagnosed by another resident (after the handover at the shift change). In one case the resident and senior physician disagreed on the final diagnosis, as the final diagnosis in the DPM was seen as a differential diagnosis by the senior physician. For further analysis concerning confidence ratings and subcategories of medical cases, we computed only completed and concordant diagnosed medical cases (*n* = 51).

The mean confidence for the final diagnoses in the DPM with 3.5 (*SD* = 0.7) was higher for the residents, compared to 3.1 (*SD* = 0.8) for the ratings of the senior physician (“+3” = *very probable* and “+4” = *practically certain*). This difference in confidence ratings resulted in a medium effect size (*Cohen’s d* = 0.53; *CI* = [−0.026–1.091]). In 17 cases (33.3 %) the confidence ratings of the final diagnoses between residents and senior physician matched perfectly. In the majority of 28 cases (54.9 %) the consensus differed only slightly with the next higher or lower rating value; whereas the maximal discrepancy comprised two scale values (out of a total of nine values) in only 6 cases (11.8 %). Overall, the senior physician was underconfident in more cases (47.1 %) than overconfident (19.6 %), compared to the confidence ratings of the residents.

### Influences of contextual factors on diagnostic process variables

Residents reported a sense of time pressure or perceived stress only in a minority of the cases traced (20 % and 15 % respectively of the 55 cases). For the number of mentioned options, searched cues, or rated confidence in the DPM group comparisons resulted only in slight differences with generally no or small effect sizes (*Cohen’s d* ranging from 0.01–0.33). DPM of residents under *time pressure* (*N* = 11) resulted in insignificantly more cues (*M* = 8.09; *SD* = 3.65 versus *M* = 7.89; *SD* = 2.73), less options (*M* = 3.18; *SD* = 1.17 versus *M* = 3.25; *SD* = 1.94), and higher confidence of the final diagnosis (*M* = 8.64; *SD* = 0.50 versus *M* = 8.48; *SD* = 0.76). DPM of residents under *stress* (*N* = 8) resulted in slightly less cues (*M* = 7.75; *SD* = 3.49 versus *M* = 7.96; *SD* = 2.83), but more options (*M* = 3.75; *SD* = 1.98 versus *M* = 3.15; *SD* = 1.78), and no difference of confidence (*M* = 8.50; *SD* = 0.53 versus *M* = 8.51; *SD* = 0.75).

The *general experience* of residents had a medium effect on the information search but not on the number of mentioned diagnoses or the confidence ratings. The more months of experience in internal medicine a resident had, the fewer cues were needed in phase 2 to verify the suspected diagnoses (*r* = −.35, *p* = 0.009, *N* = 55). *Case experience* of residents had a medium effect on the confidence rating of the final diagnosis but not on information search or the number of mentioned diagnoses. The higher the degree of similarity of a medical case a resident experienced in clinical practice, the higher confidence he or she rated for the final diagnosis (*rs* = .31, *p* = 0.039, *N* = 46).

## Discussion

A Decision Process Matrix [DPM] is a method for visualizing (assessing and mapping) individual decision-making processes. In contrast to a classical decision matrix, a DPM includes subjective confidences (instead of values) and all variables (options, cues, and confidences) are displayed in a chronological sequence. Suspected diagnoses (options), medical information (cues), and subjective confidence ratings have been assessed independently with different data collecting techniques and the matrix itself is generated in a semi-structured procedure (see Table 1). To test the feasibility of this procedure, we applied the method as a robustness test for real and heterogeneous medical cases in an emergency medicine department. In all 55 medical cases it was possible to construct a DPM under real-life conditions, although some cases were very complex. The most complex DPM was composed of 60 individual units (confidence ratings and final diagnosis included), while on average a DPM contained 27.7 individual units (*SD* = 10.6; 8–60). Hence, the contemporary assessment with the semi-structured procedure proved to be technically feasible and passed the test (*Question 1*).

While applying the method directly in a naturalistic setting, under time pressure and other uncontrollable circumstances, the internal validity of the assessed decision-making processes of physicians could only be inferred. Re-diagnoses were made independently by an expert physician on the basis of all available medical records. The concordance in the final diagnoses in 51 of the 55 medical cases (92.7 %) was satisfactorily high (*Question 2*).

To test the external validity of the procedure (*Question 3*), the structure and content of the DPM now had to be compared with the theoretical assumptions and results from the review of the literature. Once again we want to emphasize that a DPM in itself is a method of assessing and mapping individual decision-making processes, and is not a model for testing or explaining those processes. Therefore, when compared to the literature, correspondences, discrepancies, or additional aspects should be reported and discussed. For example, the common described process of hypothesis testing should be reflected in most of the DPM.

### Phases of decision-making processes

Within the DPM, a consistent behavioral pattern consisting of three distinct phases was found: The first phase followed the sequence cue(s) – mentioned option – corresponding confidence(s) at the beginning of the diagnostic process. The second phase followed at least once or repeatedly the sequence (single) cue – confidence (according to that cue and one of the specific options mentioned before), whereas the third phase denoted the final diagnosis (see example in Fig. 1).

The first phase could be identified as the option generation stage, and the second phase corresponded to the stage of option verification. The third phase completed the diagnostic process with the determination of the final decision with one final diagnosis, or one or more differential diagnoses. Based on this final decision, the appropriate treatment was initiated. Accordingly, just a few cues led to the generation of one or more suspected diagnoses, which were then tested for plausibility with additional information [5]. Sometimes further suspected diagnoses emerged during this second verification stage [31].

The present behavioral pattern of the residents during the diagnostic process fits very well with the framework of Betsch and his colleagues [9]. While phase 1 corresponds to the *generation of options*, and phase 2 to *information search*, phase 3 (decision-making for final diagnosis) is in line with Betsch et al’s *evaluation and decision-making process*. In our setting, the antecedent step, *identifying the decision-relevant situation,* is trivial, because patients seek medical help, whereas the two subsequent steps, *determining the most appropriate course of action and feedback,* requires implementing and monitoring the requisite therapy. The approach of Baerheim [8] also focuses on phase 1 and 2, but does not include the third phase. In addition to the model of Baerheim, we consider phase 3 (deciding on the final diagnosis) to be an important and independent phase. In this phase, the search for further cues and options is halted, and the final diagnosis is determined. As a consequence, all further reasoning and action will be based on this determination. Therefore, phase 3 may be regarded as the transition from diagnostic reasoning to treatment initiation. Furthermore, within the framework of Gigerenzer and colleagues [32], phase 3 corresponds to the third building block (decision rule) of process models under conditions of uncertainty. Therefore, we conclude that the medical diagnostic process consists of three stages: first, a stage of *option generation*; second, a stage of *option verification* and, third, the stage where the *final diagnosis is determined*.

Considering these three stages found in the DPM, the entire diagnostic process might be regarded as a unique hypothetico-deductive approach [1]. However, when looking at stage one in more detail, pattern recognition might be regarded as an underlying process for generating options. When looking at stage two, one might conclude that the prediction rule is one way to test the suspected diagnoses. Hence, it is more probable that all three models (pattern recognition, prediction rules and hypothesis testing) may occur within the same diagnostic process but at different points in time. Further investigations are needed to clarify the overlap and interaction between these three models. Since additional suspected diagnoses (28 % of all mentioned options) were also generated during the option testing phase, simple decision-making models fall short of an adequate description of the dynamic process. Diagnostic decision-making in a naturalistic setting, as in the emergency medicine department, appears to be much more dynamic and complex.

### The role of confidence ratings during decision-making processes

Subjective confidence played an important role during the entire diagnostic process, as the physician aimed to confirm suspected diagnoses (observing increasing confidence [11]) or to exclude them (resulting in decreasing confidence).

In stage 2 (option verification), subjective confidence changes in the direction of the two desired states (inclusion or exclusion); therefore, this diagnostic process can be regarded as an evidence accumulation process [15, 16]. Physicians strive for high confidence in both the verification as well as the exclusion of suspected diagnoses. Two-thirds of those final diagnoses were rated with the highest possible value (+4 = *practically certain*), whereas more than half of the discontinued diagnoses were given the lowest possible value (−4 = *practically impossible*). However, the actual reason for terminating the information search process (stage 2) must remain open [16–18]. Another open question is the preferred search strategy in stage 2. When looking at the matrices, simple strategies like “Elimination by aspects” [33] or “Satisficing” [34] seem to be exceptions, for example, ruling out red flag signs of a potentially fatal disease. With the help of DPM one should be able to have a more detailed look at individual strategy preferences in future studies [35].

As far as we can see in the DPM physicians did not focus on individual information units when rating confidence during the decision-making process. In fact, physicians rated each confidence in the sense of overall confidence, which means that he or she indicated the certainty for or against a specific option at that specific point in time. This observed continuously aggregation of previously collected information in the meaning of overall confidence speaks again for an evidence accumulation [15] approach in medical decision-making.

Regarding stage 3, the accuracy of the final diagnoses was verified by a senior physician and was concordant in more than 90 % of the cases. Interestingly, the senior physician was systematically underconfident (with a medium effect size). This underconfidence might be seen as a strategy of caution, because the diagnoses of the senior physician were only based on the medical records, including laboratory and imaging tests without having had direct patient contact.

### The potential role of intuition and influence of contextual factors

It is remarkable that almost 90 % of the final diagnoses were already mentioned as suspected diagnoses in stage one [5]. Several researchers give intuition great importance for the early recognition of suspected diagnoses [14, 36, 37]. This might be due to pattern recognition combined with underlying intuitive processes in stage 1 (associative or matching intuition [38]). With future studies and the help of DPM it should be possible to reveal and locate potential intuitive aspects of medical decision-making.

Contextual factors such as the perceived time pressure or stress had a negligible influence on the diagnostic process. However, more experienced physicians needed fewer cues to verify suspected diagnoses, which was expected. Apart from general or case experience, one can conclude that the DPM in our study are not influenced by contextual factors (*Question 4*). But we cannot rule out the fact that including more medical cases could have showed significant results for those control variables.

### Limitations and methodological potential

Overall one can conclude that we managed to assess the DPM in our study (under the given circumstances) in a more or less valid way. With the chosen semi-structured procedure it is very likely that all mentioned options have been included. One major challenge remains the real-time capturing of emerging suspected diagnoses in the memory of the physician. When constructing a DPM just from the physician’s notes and memory, a certain recall bias cannot be ruled out, while a real-time tracing of suspected diagnoses by interrupting the taking of the patient’s history would have disturbed the patient-physician relationship.

With the student’s note-taking throughout the process and the semi-structured procedure it is also conceivable that almost all of the relevant medical information has been assessed. An unresolved challenge remains the role played by the concept of relevant or meaningful information and how this relevant information (cue) is defined or comprised of single information fragments. In our study, single questions or cues were automatically merged into meaningful informational units by the student and then verified and approved by the physician.

Probably the major weak point in our chosen design is the subjective confidence ratings. As probabilities are rated promptly after the discharge of the patient a certain recall bias cannot be ruled out. So all results about the confidence ratings have to be interpreted carefully. Furthermore and in general, there is no validated measuring scale of probabilities (rated confidence). Confidence scales with verbal labels are not distributed equidistantly, and numerical probabilities are more difficult to elicit and understand [39, 40]. These aspects require further exploration or validation in the future.

Assessing a DPM in its entirety requires sophisticated methods. It is a challenge to record all three variables (options, cues, and confidences) at the same time as validly and promptly as possible. Depending on the research questions and proceeding as true to life as possible, a researcher has to prioritize during the process, for example as we did in recording the confidence ratings in a final step. But there are several ways to assess a DPM in a reduced manner. In a *confidence profile* for example, one can assess and display all mentioned options and their corresponding confidence ratings at a specific time point during the decision-making process. This recording can be repeated at a second time point for all aforementioned options and newly emerging options. While totally ignoring underlying information (cues), with *confidence profiles* one can therefore assess and map the course of confidence for all mentioned options in a more direct way. Furthermore, the use of electronic devices such as audio pens or video recordings of confidence ratings or suspected diagnoses that are verbally articulated and immediately written down might be very helpful in the future.

### Implications for research and clinical practice

Even though the construction of a DPM can become quite extensive, the possibilities for analysis and testing hypotheses are numerous. For further validation of the DPM or for testing specific process models, separate studies under laboratory conditions with case vignettes should be favored. And last but not least, this method of assessing DPM is not limited to medical decision-making or clinical problem solving.

A DPM is a dense mapping of a diagnostic process and offers many possibilities for individual feedback about the most important aspects of the process. From an educational perspective, a reanalysis of a DPM with the help of a supervisor might result in improved diagnostic skills [6]. For example, when considering the relevant cues, one might focus on the accuracy of the options mentioned, the optimization of search strategies and the obtaining of feedback about the certainty of suspected diagnoses at different points in time, etc. For example, DPM allow the addressing of specific questions like: *Why was a suspected diagnosis not considered earlier in the process? Why was the physician overconfident or underconfident about a specific diagnosis at a specific point in time? Why did he or she miss a diagnosis? Did he or she interpret the specific cues correctly?* Being aware of and communicating the subjective confidences in suspected diagnoses might serve as a uniform currency that helps to avoid unnecessary steps, scrutinizes possible heuristic biases, and enhances the overall quality control. In certain cases, a satisficing strategy could be optimal; on the one hand, testing strong suspected diagnoses sequentially and defining case-specific confidence thresholds for inclusion as well as for exclusion may be more productive. Furthermore, incorporating intuition might result in a shortening of the diagnostic process, as 90 % of the final diagnoses were mentioned already as suspected diagnoses during stage one [41].

Our study ties in with the seminal work of Elstein and colleagues [5], with their fundamental question “… of how humans, with a vastly different information-processing capability, in fact perform this task” (p. 3) of medical problem solving. But beyond past and current conducted experimental studies with mostly medical “paper problems” [5] such as presented descriptions or simulations of virtually constructed medical cases, DPM as a new methodological tool allow for the mapping of individual diagnostic processes in real life with only minimal disturbance of the naturalistic process itself. Recording DPM is a consequential implementation of several different process-tracing techniques (e.g., recording observable verbal and non-verbal behavior of social interactions as well as concepts from memory, e.g., suspected diagnosis) [25].

## Conclusions

The Decision Process Matrix [DPM] is a valuable methodological tool for tracing real and individual diagnostic processes of physicians. DPM map the most important aspects of decision-making processes (like suspected diagnoses, information and subjective confidence ratings, all with a consecutive numbering of occurrence). In medical decision-making, a consistent three-phase pattern was revealed, consisting of: 1) option generation, 2) option verification, and 3) determination of the final diagnosis. Confidence ratings of the physicians reflect the diagnostic process: for the final diagnoses the confidence increased over all three phases, while for the finally excluded suspected diagnoses the confidence decreased continuously. The methodological approach with DPM allows further investigations into both the underlying cognitive diagnostic processes on a theoretical level and the improvement and streamlining of individual clinical reasoning skills in practice.

## Abbreviations

DPM: Decision process matrix
ED: Emergency department
FD/DD: Final diagnosis and/or specific differential diagnoses
I&LT: Ordering of imaging and laboratory tests
MHT: Medical history-taking
PE: Physical examination
SD: Standard deviation
